# Expression of *MRP1* gene in acute leukemia

**DOI:** 10.1590/S1516-31802008000300007

**Published:** 2008-05-01

**Authors:** Frouzandeh Mahjoubi, Masoud Golalipour, Ardeshir Ghavamzadeh, Kamran Alimoghaddam

**Keywords:** Multidrug resistance, Chemotherapy, Polymerase chain reaction, Leukemia, myeloid, Leukemia, lymphoblastic, acute, Resistência a múltiplas drogas, Quimioterapia, Reação em cadeia da polimerase via transcriptase reversa, Leucemia mielóide, Leucemia linfocítica aguda

## Abstract

**CONTEXT AND OBJECTIVE:**

Overexpression of the multidrug resistance-associated protein 1 (*MRP1*) gene has been linked with resistance to chemotherapy *in vitro*, but little is known about its clinical impact on acute leukemia patients. Our aim was to investigate the possible association between *MRP1* gene expression level and clinical outcomes among Iranian leukemia patients.

**DESIGN AND SETTING:**

This was an analytical cross-sectional study on patients referred to the Hematology, Oncology and Stem Cell Research Center, Sharyatee Public Hospital, whose diagnosis was acute myelogenous leukemia (AML) or acute lymphoblastic leukemia (ALL). All molecular work was performed at NIGEB (public institution).

**METHODS:**

To correlate with prognostic markers and the clinical outcome of acute leukemia, *MRP1* gene expression was assessed in 35 AML cases and 17 ALL cases, using the quantitative real-time polymerase chain reaction and comparing this to the chemotherapy response type.

**RESULTS:**

Mean expression in AML patients in complete remission (0.032 ± 0.031) was significantly lower than in relapsed cases (0.422 ± 0.297). In contrast, no significant difference in *MRP1* mRNA level was observed between complete remission and relapsed ALL patients. There was a difference in *MRP1* expression between patients with unfavorable and favorable cytogenetic prognosis (0.670 ± 0.074 and 0.028 ± 0.013, respectively). *MRP1* expression in M5 was significantly higher (p-value = 0.001) than in other subtypes.

**CONCLUSIONS:**

The findings suggest that high *MRP1* expression was associated with poor clinical outcome and was correlated with the M5 subtype and poor cytogenetic subgroups among AML patients but not among ALL patients.

## RESUMO

**CONTEXTO E OBJETIVO:**

A superexpressão do gene de resistência a múltiplas drogas associado à proteína 1 (*MRP1*) tem sido ligada à resistência à quimioterapia *in vitro*, porém pouco é conhecido sobre seu impacto clínico nos pacientes com leucemia aguda. Nosso objetivo foi investigar a possível associação entre a expressão do gene *MRP1* e os desfechos clínicos em pacientes iranianos com leucemia.

**DESENHO E LOCAL:**

Este foi um estudo analítico transversal em pacientes encaminhados ao Centro de Pesquisa em Hematologia, Oncologia e Células Tronco do Hospital Público de Sharyatee, com diagnóstico de leucemia mielóide aguda (LMA) ou leucemia linfoblástica aguda (LLA). Todo trabalho molecular foi realizado no NIGEB (instituição pública).

**MÉTODOS:**

Para correlação de marcadores prognósticos e desfechos clínicos da leucemia aguda, a expressão do *MRP1* foi avaliada em 35 casos de LMA e 17 de LLA, usando a reação da cadeia de polimerase quantitativa em tempo real, e comparando este dado ao tipo de resposta à quimioterapia.

**RESULTADOS:**

A média da expressão em pacientes com LMA em remissão completa (0,032 ± 0,031) foi significativamente menor que aquela dos casos recidivantes (0,422 ± 0,297). Por outro lado, não foram observadas diferenças significativas nos níveis de mRNA para *MRP1* entre os casos de LLA com remissão completa e os casos recidivantes. Houve uma diferença na expressão de *MRP1* entre pacientes com prognóstico citogenético não-favorável e favorável (0,670 ± 0,074 e 0,028 ± 0,013, respectivamente). A expressão de *MRP1* em M5 foi significativamente maior (valor de p = 0,001) do que em outros subtipos.

**CONCLUSÕES:**

Os achados sugerem que a alta expressão de *MRP1* se associou com o pior desfecho clínico, estando correlacionada com o subtipo M5 e os subgrupos citogenéticos menos favoráveis para os pacientes com LMA, mas não para pacientes com LLA.

## INTRODUCTION

The multidrug resistance (MDR) phenotype in some cancers, especially leukemia, remains a major problem in chemotherapy treatment.^1^ Although new drugs and treatment protocols have improved the disease prognosis among leukemia patients, initially responsive tumors ultimately relapse and develop resistance to the drugs.^2^ When leukemic cells become resistant to an antineoplastic agent, treatment becomes very difficult because the range of resistance generally extends even to drugs to which leukemic cells have never been exposed.^1^

Several mechanisms for MDR have been identified. One of the major mechanisms for drug resistance is associated with altered anticancer drug transport, mediated by members of the human-adenosine triphosphate binding cassette (ABC) transporter superfamily proteins.^3,4^ These transporters are capable of decreasing the intracellular drug concentration *in vitro*.^5^ One of the best known multidrug resistance genes is the multidrug resistance gene, multidrug resistance-associated protein 1 (*MDR1*), which is located on chromosome 7 and codes a p-glycoprotein (Pgp). The role of Pgp in inducing MDR has been confirmed by *in vitro* studies.^6^ However, trial usage of Pgp modulators in leukemia has produced controversial results7 and it seems that *MDR1* function in acute myelogenous leukemia (AML) patients does not correspond to *in vitro* drug resistance.^8^ In several studies on drug-resistant cell lines with increased drug efflux, no significant *MDR1* expression was observed.^9^ In addition, the kinetics of anthracycline transport by *MDR1* and *MRP1* are very similar.^10^ This suggests that alternative proteins, such as *MRP1*, may play a role in the MDR phenotype.^11^

The *MRP1* gene is a member of the ABC-transporter superfamily of membrane drug transporter genes, located on chromosome 16p13. Its protein product has been shown to transport chemicals conjugated with sulfate, glutathione or glucuronate and various other organic anions.^12^ Several studies have shown that *MRP1* expression confers *in vitro* resistance to a wide range of anticancer drugs, such as anthracyclines, vincristine and epipodophyllotoxins.^13^ However, the role of *MRP1* in inducing the MDR phenotype in cancer patients is still controversial.^14^ These differences are partly due to the variety of detection methods employed, as well as the definition of overexpression used.^15^ Nevertheless, from a pharmacological viewpoint, it is important to determine whether or not the expression of *MRP1* may change with the disease state and hence whether the expression of Pgp affects the clinical outcome. Relevant investigations published over the last few years have used different methods such as Northern blot,^16^ dye exclusion,^14^ flow cytometry^9,17^ and reverse-transcriptase polymerase chain reaction (PCR)^17^ to detect *MRP1* expression in cell line samples.^14^ However, these assays have either proven difficult to standardize or tedious to perform. Very importantly, all of them are semiquantitative and therefore the amount of expression cannot be expressed as definite values.

## OBJECTIVE

In this study, we aimed to investigate the expression of *MRP1* in leukemia patients at transcription level and examined whether the messenger RNA (mRNA) level of *MRP1* was higher in relapsed patients who were still responsive to drugs (Relapsed) and relapsed patients who presented no response to drugs (NR), compared to control groups (patients at complete remission, CR) and also to healthy individuals.

## MATERIALS AND METHODS

### Patients and samples

Peripheral blood (PB) and bone marrow (BM) aspirates from 52 patients, including 35 patients with AML and 17 patients with ALL, were collected. The samples were collected from patients who were at the diagnosis, remission or relapse stage. For some of the patients, *MRP1* expression evaluations could be followed through the course of the treatment or at regular check-ups. The patients’ ages were from 15 to 50+ years (mean = 35). Two patients were classified with M0, four patients with M1, seven patients with M2, six patients with M3, seven patients with M4 and eleven patients with M5. Five patients were classified with L1 and twelve patients with L2. The white blood cell (WBC) count ranged from 3,500 to 200,000. The majority of the patients were CD34 negative (n = 35).

For each patient, several clinical and pathological characteristics including age, sex, white and red blood cell counts at diagnosis, leukemia FAB (French-American-British) subtype, CD34 expression and karyotype were considered. The patients were divided into three groups: CR (complete remission), Relapsed and NR (no response). The response to treatment was classified as described previously.^18^ Patients were considered to be in the CR group if the criteria established were met, including cellular marrow with < 5% blast cells, neutrophil count > 1.5 × 109/l, platelet count ≥ 100 × 109/l and no evidence of leukemia at other sites observed within six months. The NR group included patients with cellular marrow > 5% blast cells, or evidence of leukemia at other sites. Finally, the Relapsed group consisted of patients who presented a relapse within six months after remission. The resistant HL-60 cell line, which is known to overexpress *MRP1* (generously provided by Dr. Dizage), and peripheral blood from 10 healthy individuals were used, respectively, as overexpression and normal controls.

### Total RNA isolation and complementary DNA (cDNA) synthesis

Leukemic blasts from PB and BM samples were separated by Ficoll-Paque Plus® density gradient centrifugation, in accordance with the manufacturer’s instruction (Amersham Biosciences), and then suspended in phosphate-buffer saline (PBS). Total RNA was isolated from lymphocytes using the TRIZOL reagent (Gibco BRL), in accordance with the manufacturer’s protocol. Pelleted RNA was resuspended in extragene E solution. Its concentration was determined by spectrophotometry and its purity was assessed in relation to an OD_260_/OD_280_ (optical density) absorption ratio greater than 1.7. RNA was stored at -80 °C until use. One μg of total RNA from each sample was used to synthesize first-strand cDNA. The RNA was incubated for one hour at 42 °C in a 20 μl of RT buffer containing 100 units of Moloney Murine Leukemia Virus (MMLV) reverse transcriptase, 20 units of RNasin, 1 mm of each dNTP and random hexamer primer (all from Promega). The resulting cDNA was diluted in diethylpyrocarbonate (DEPC) treated pure water and was used in the real-time PCR reaction.

### Real-time polymerase chain reaction

The sequences of primers for assessing *MRP1* expression were as follows: forward 5´ - CGG AAA CCA TCC ACG ACC CTA ATC - 3´ and reverse 5´ - ACC TCC TCA TTC GCA TCC ACC TGG - 3´. The sequences of primers for assessing β*2M* (β-2-microglobulin) expression were: forward 5´ - CTA TCC AGC GTA CTC CAA AG - 3´ and reverse 5´ - GAC AAG TCT GAA TGC TCC AC - 3´. The primers were designed using Primer Premier 5.0 software and synthesized by MWG Biotech AG. All primer sequences were checked by means of the alternative splicing electronic real-time PCR (ASePCR) program (http://genome.ewha.ac.kr/ASePCR) for absence of any false priming sites. The length of the amplicon was 294 bp for *MRP1* and 147 bp for *β2M*.

To quantify the gene expression, we used the Lightcycler^TM^ system (Roche Applied Sciences) and the Fast-Start DNA Master SYBR-Green I kit (Roche Applied Sciences). All reactions were carried out in a total volume of 20 μl in capillary tubes. Each reaction mix contained 0.6 μm of each primer, 2.5 mM MgCl_2_ and 2 μl of Fast-Start Master solution. A total of 18 μl of this reaction mix was placed into glass capillaries and 2 μl of cDNA (based on 1 μg total RNA) was added as the template. The capillary tubes were capped, centrifuged (2500 rpm, 1 second) and placed in the carousel under reduced light conditions. The PCR conditions were optimized with regard to primer and MgCl_2_ concentrations and annealing temperatures.

A standard Lightcycler PCR program was established for each gene. The thermal cycling consisted of an initial denaturation step at 95 °C for 10 minutes followed by a three-step (primer annealing, amplification and quantification) program repeated for 50 cycles with temperature ramp rate of 20 °C/second. The program consisted of 95 °C for 1 second, 64 °C for 10 seconds and 72 °C for 40 seconds with single fluorescence acquisition at the end of the elongation step. The third segment consisted of a melting curve program at 95 °C for 0 seconds, 72 °C for 10 seconds and 95 °C for 0 seconds with a liner temperature transition rate of 0.1 °C/seconds with continuous fluorescence acquisition. Finally, a cooling program cooled the reaction mixture to 40 °C. The β-2-microglobulin PCR program was the same except that the annealing temperature in the second segment was 50 °C for 10 seconds. To ascertain that the fluorescence signals were associated with specific products, melting curves for each reaction analyzed and the PCR products were checked on 1.5% agarose gel electrophoresis for the absence of nonspecific bands.

### Cytogenetic analysis

Cytogenetic studies on nonstimulated bone marrow samples were performed using a standard protocol. Bone marrow cells were cultured for 24 and 48 hours in RPMI 1640 (Roswell Park Memorial Institute) culturing medium with 10% fetal calf serum (FCS). The cultures were harvested and 20 banded metaphases were analyzed. The results were described in accordance with the International System for Human Cytogenetic Nomenclature.^19^

### Data analysis

The raw data were analyzed using version 3.03 of the Lightcycler software. The crossing point was defined as the cycle number at which the fit line in the log-linear portion of the plot intersected the threshold level. An external standard curve for *MRP1* and β*2M* was generated from serial dilution of mRNA of each gene. The standard curve was constructed from the plot of crossing points against the copy number of serially diluted standard samples. For each sample, the amounts of *MRP1* and the housekeeping gene were measured. Finally, the relative copy number was calculated as the ratio of *MRP1* to β*2M* copy number in each sample.

Statistical calculations and tests were performed using the Statistical Package for the Social Sciences (SPSS) 13.0 software (SPSS, Inc., Chicago, United States). The normality of the data was tested using the Shapiro-Wilk normality test. Differences between groups were analyzed using the one-way analysis of variance (ANOVA) test and correlations between clinical characteristics and expression levels were determined using Pearson’s chi-squared test. The statistical significance limit was defined as p ≤ 0.05.

## RESULTS

### Real-time polymerase chain reaction validation

**Figure 1:**
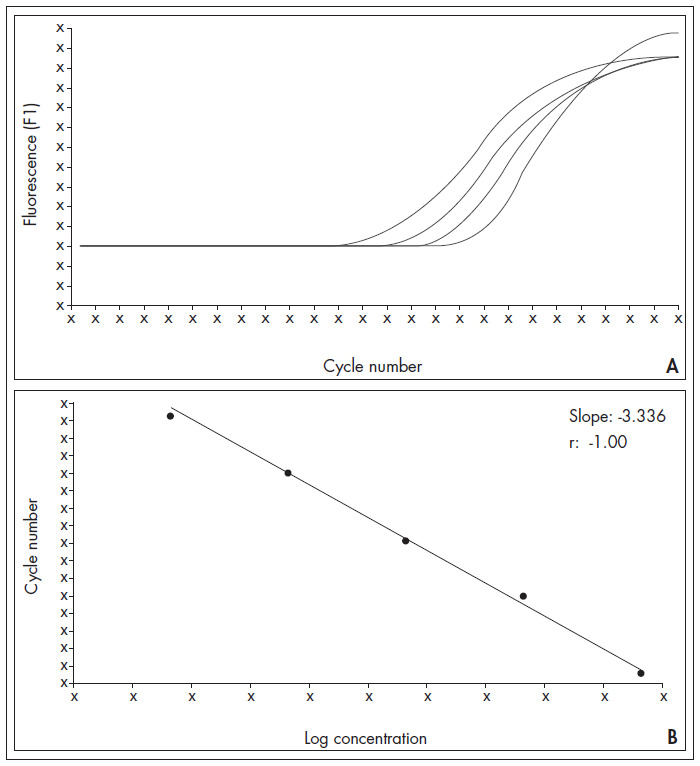
Amplification (A) and calibration curve (B) for serial dilutions of multidrug resistance-associated protein 1 (*MRP1*). The calibration curve shows a good correlation between transcript copy number and threshold cycle (r = -1.00). The calibration curve slope (-3.336) is close to the optimal curve slope and represents high polymerase chain reaction (PCR) efficiency (0.98).

The real-time PCR products showed only one band of the expected size upon electrophoresis and only a single melting temperature peak was observed for each reaction, thus suggesting that nonspecific amplification did not occur. To establish optimal conditions for quantitative analysis, a calibration curve was prepared using serial dilutions of *MRP1* RNA (Figure 1A). The calibration curve showed a good correlation between transcript copy number and threshold cycle (r = -1.00). To ensure high PCR efficiency, we tried to reach a calibration curve slope near to -3.322 (optimum curve slope) and y-intercepts close to the C_t_ value of the negative control. Our assay was linear from 6 × 105 copies to 6 × 1010 copies (Figure 1B).

### Determination of cutoff values

**Figure 2:**
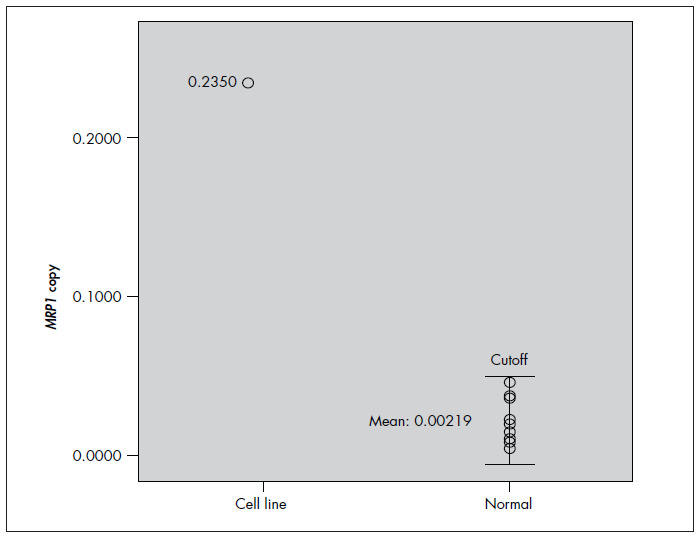
Expression of multidrug resistance-associated protein 1 (*MRP1*) messenger RNA (mRNA) in peripheral blood of healthy individuals and the HL-60 cell line. The copy number of *MRP1* is expressed as the ratio of *MRP1* to β*2M* copy number (each circle represents a sample). In accordance with *MRP1* expression in healthy individuals, the cutoff is defined as mean ± 2 standard deviations (SD). The HL-60 resistant cell line showed *MRP1* expression levels higher than the cutoff.

To obtain a cutoff value to discriminate between normal and upregulated states of samples, we examined the expression of *MRP1* mRNA in the cell line and healthy blood by means of real-time PCR. The final results were expressed as the ratio of *MRP1* to β*2M* copy number in each sample. The mean *MRP1* expression was 0.235 in resistant HL-60 but was 0.0219 in healthy samples (Figure 2). On the basis of the *MRP1* expression value in healthy samples, we defined the cutoff for *MRP1* as 0.0575 (mean ± 2 standard deviations, SD). Therefore, all values above this cutoff were assumed to represent overexpression.

As a second approach, we defined an alternative cutoff value based on the *MRP1* expression level in newly diagnosed cases. According to the mean expression level in newly diagnosed patients, we assumed 0.0636 and 0.1262 as alternative cutoff values for AML and ALL, respectively.

### Expression of MRP1 in relapsed and non-response patients

**Figure 3:**
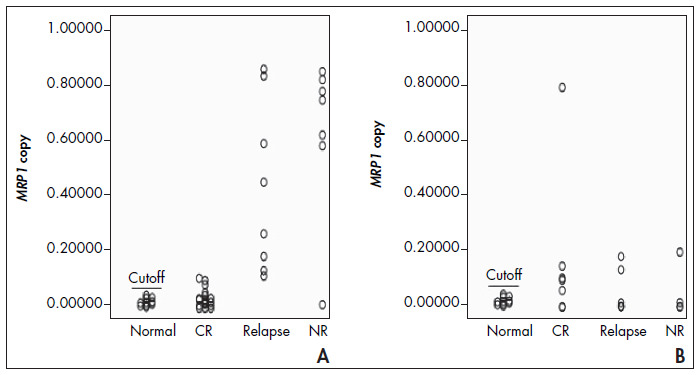
The expression of multidrug resistance-associated protein 1 (*MRP1*) messenger RNA (mRNA) at onset and relapse in cases of acute myelogenous leukemia (AML) (A) and acute lymphoblastic leukemia (ALL) (B) is shown (each circle represents a sample). In AML, the distribution of copy numbers in CR and healthy individuals is different from the distribution in relapsed patients. Patients in the NR group (except one) show higher messenger RNA (mRNA) levels than do relapsed patients. In contrast, mRNA levels in ALL patients have similar distributions in the different groups.

A statistically significant (p < 0.05) increase in *MRP1* expression was observed when samples from the Relapsed and NR groups of AML patients were compared with samples from patients with CR (Table 1). *MRP1* overexpression was observed in all AML cases at relapse and in all but one NR patients (Figure 3A). The mean expression in the CR group (0.032 ± 0.031) was significantly lower than the mean expression in the Relapsed group (0.422 ± 0.297) and NR group (0.619 ± 0.284). Table 2 shows the mean difference between normal and AML samples and their p-values. Although no significant difference was observed between NR and Relapsed patients (p = 0.171), the *MRP1* expression level in both groups was meaningfully (p < 0.005) different from the Healthy or CR groups and there appeared to be a tendency towards higher *MRP1* expression at the time of relapse.

**Table 1:** Correlation between multidrug resistance-associated protein 1 (*MRP1*) expression and clinical characteristics of acute myelogenous leukemia (AML) and acute lymphoblastic leukemia (ALL) patients according to different cutoffs. The p-values for AML patients suggest that the French-American-British (FAB) subtype and cytogenetic risk group are correlated with *MRP1* expression level (0.017 and 0.042, respectively). In contrast, the p-values for ALL patients show no correlation between *MRP1* expression and clinical findings. The alternative cutoff makes the p-values different but no significant changes are observed in the final results

Disease		Clinical characteristics						
		Gender	Age	FAB subtype	White blood cell (WBC) count	CD34 expression	Cytogenetic group	Clinical response
Normal	ALL	0.084	0.126	0.433	0.281	0.624	0.988	0.2210
cutoff	AML	0.496	0.929	0.017*	0.586	0.485	0.042*	0.0010*
Alternative	ALL	0.232	0.130	0.149	0.281	0.415	0.266	0.6230
cutoff	AML	0.621	0.797	0.012*	0.589	0.494	0.021*	0.0002*

* *The mean difference is significant at the 0.05 level.*

**Table 2:** Multidrug resistance-associated protein 1 (*MRP1*) expression level in acute myelogenous leukemia (AML) and acute lymphoblastic leukemia (ALL) samples. Expression of *MRP1* in the nonresponse (NR) group (phase i) compared with normal, complete remission (CR) and early relapsed groups (phase j). In ALL samples, no significant difference was observed. In contrast, in AML samples from relapsed and NR groups, the *MRP1* level is significantly different from the healthy and CR groups (p-value = 0.001), but the mean difference between the NR and Relapsed patients is nonsignificant (p-value = 0.171)

Phase (j)	Phase (i)	Mean difference (i-j)	Standard error	p-value
ALL	Normal	0.0317	0.0885	0.988
	CR	0.1160	0.0938	0.680
NR	Relapsed	0.0022	0.0966	1.000
AML	Normal	0.6003*	0.0817	0.001
	CR	0.5874*	0.0728	0.001
NR	Relapsed	0.1969	0.0858	0.171

* Significant at the 0.01 level.

Among the 17 ALL cases studied, eight cases showed overexpression of *MRP1*, including five CR, two Relapsed and one NR patient (Figure 3B). In contrast, when ALL cases were analyzed according to the second cutoff value, only four cases showed overexpression (one CR, one NR and two Relapsed). The difference in mean expression was not significant between CR and Relapsed (0.169 ± 0.275 compared with 0.055 ± 0.079; p = 0.608) or CR and NR (0.169 ± 0.275 compared with 0.053 ± 0.095; p = 0.680)

### Correlation of MRP1 expression with other clinical characteristics

The correlations of other well-known variables such as gender, age, WBC count at diagnosis, CD34 expression, FAB subtype and cytogenetic findings with *MRP1* mRNA copy number was also analyzed. There was no clear relationship between *MRP1* expression and gender, age, WBC count and CD34 expression (Table 1). However, cytogenetic group and FAB subtype were two main factors that were shown to correlate with *MRP1* transcript level in both ALL and AML patients.

The patients were assigned to different genetic subgroups according to the chromosomal abnormalities identified in the leukemic cells and the findings were described in accordance with the international nomenclature.^20^ We defined abnormalities such as inv16, t(15;17), t(8;21) and deletion 16 as favorable normal cytogenetic prognostic categories, +8 as intermediate and -5/del, -7/del and 11q2.3 as unfavorable. Abnormalities associated with AML were found in 11 cases including t(8;21), inv(16), t(15;17), del 7, del 11q2.3, t(12;22), deletion 16 and trisomy 8. According to this scheme, we found that there was a difference in *MRP1* expression between patients with unfavorable and favorable cytogenetic prognoses (0.670 ± 0.074 and 0.028 ± 0.013, respectively), and the mean difference between two groups was 0.642 ± 0.183 (p-value = 0.006). Interestingly, we found one patient in the NR group with no apparently chromosomal abnormality whose *MRP1* expression was lower than the cutoff. Four patients with favorable cytogenetic results had no *MRP1* expression. Three of those cases had inv16 and one case had del 16, and all these four cases were in the CR group.

There was also heterogeneity of *MRP1* expression among AML patients with different FAB subtypes. The AML patients presented six subtypes, including M0 to M5. According to our data, the mean expression of *MRP1* in the M5 subtype (0.6415 ± 0.071) was significantly higher (p-value = 0.001) than in the other subtypes. In contrast, normal *MRP1* expression was relatively more frequent in M0, M1 and M3 cases (100%, 50% and 66%, respectively) and less frequent in M2, M4 and M5 (28%, 0% and 0%).

According to the alternative cutoff, the p-values in ALL and AML cases underwent little modification. The overall result did not change, although greater significance was observed in correlations between mRNA levels and cytogenetic risk group or FAB subtype (Table 1).

## DISCUSSION

Although the antineoplastic drugs currently available are effective for treating various cancers, they may prove to be ineffective in treating some primary or relapsed types of neoplasia. Multidrug resistance is seen to be the most significant barrier to effective treatment of malignant tumors in general. Identification of variables that might be used to predict the response of tumors to treatment is constantly under discussion in oncology. Several genes are thought to participate in the MDR phenotype. Because many kinds of antileukemic agents can be substrates for the efflux pumps, upregulation of these pumps leads to insufficient concentrations of agents in cancer cells, even when used at maximum dosages.

The best-known resistance gene in leukemia patients that has been shown to be correlated to poor outcomes is mediated by the *MDR1* gene.^3,6^ However, clinical investigations into the role of *MDR1* have yielded inconsistent results that are difficult to interpret.^14^ In several papers, the expression of Pgp did not correlate with the multidrug resistance phenotype.^9,21^ Thus, alternative proteins such as *MRP1* may also contribute towards resistance in acute leukemia cases. Although in several studies *MRP1* has been shown to be involved in multidrug resistance,^22,23^ in other investigations *MRP1* expression appeared to have no impact on treatment outcome in AML cases.^24-26^ *MRP1* has been detected in a wide variety of solid and hematological tumors, but evaluation of the presence of *MRP1* protein or its cognate mRNA in a tumor sample is complicated by the fact that the *MRP1* gene is expressed in the tissue from which these tumors originate and the protein is also expressed in all lineages of normal hematopoietic cells. Thus, the clinical impact of *MRP1* expression remains controversial. In addition, one of the main reasons for the contradictory results from studies on drug resistance relates to methodology, in addition to differences in the definition of overexpression.^15^

Lack of adequate quantitative methods for assessing the amount of transcription is the most important problem making the data hard to interpret. 3-(4,5-dimethylthiazol-2-Yl)-2,5-diphenyltetrazolium bromide (MTT) assaying is a widely used and validated method for analyzing *in vitro* drug resistance, but it may not be useful for detecting resistance induced by ABC transporters. This is partly because the leukemia cells are heterogeneous.^27^ The flow cytometry method can overcome the heterogeneity of leukemic cells by targeting leukemic cells with specific antibodies.^9,17^ However, even with secondary antibodies, flow cytometry sensitivity is very low. Northern blot assaying is simple to perform, but the sensitivity of this technique is the lowest of all the methods described for mRNA quantification.^16^ Competitive reverse-transcriptase PCR assaying, which is widely used for mRNA quantification, is correlated with a high risk of contamination because it needs post-PCR treatments.^17^ In addition, this method is semiquantitative and only yields data discontinuously, such that the cutoff cannot be determined. All of the methods described are difficult to standardize and hard to perform. In contrast, real-time PCR is simple to use, and requires only minimal experience and skills. It provides maximum sensitivity (e.g. 10,000 times more sensitive than northern blot) and is the only method that allows quantification.

In many studies, real-time PCR has been widely used to quantify ABC transporter mRNA levels in different cancer cells. In these studies, *MRP1* expression did not correlate strongly with clinical resistance in leukemia patients. Fujimaki et al. used real-time PCR to determine *MDR1* and *MRP1* transcript level impact in AML patients. They found that increased *MDR1* but not *MRP1* expression at diagnosis correlated with the multidrug resistance phenotype.23 Considering the molecular aspect of gene expression regulation, the *MDR1* expression in leukemic cells exposed to antineoplastic agents is regulated by two distinct processes: stabilization of messenger RNA and initiation of the translation process. *In vitro* study of these phenomena has revealed that *MDR1* overexpression does not always occur via the activation of transcription.28 In contrast, *MRP1* protein levels increase according to *MRP1* mRNA levels *in vivo*.29 The short-lived *MDR1* mRNA of naive cells (not exposed to drugs) is stabilized (half-life greater than 10 hours) following short-term drug exposure. However, this stabilized mRNA has not been associated with polyribosome translation and does not direct Pgp synthesis.28 Thus, *MDR1* mRNA levels do not necessarily correlate with P-glycoprotein expression, and measuring *MDR1* mRNA as a clinical surrogate for Pgp-mediated drug resistance is inappropriate.

Furthermore, identification of a valid reference gene for data normalization remains the most stubborn of the problems in quantitative PCR. *GAPDH* and β*-Actin* genes continue to be utilized as normalizers despite continuing reports that emphasize the problems associated with their use.^30^ It is now well documented that *GAPDH* and β*-Actin* mRNA levels are not constant, even in cellular subpopulations of the same pathological origin.^31^

To overcome this problem, we have designed and validated a real-time PCR assay to monitor *MRP1* transcript levels in acute leukemia at different stages of the disease. We used β*2M* because it has no pseudogene and the use of β*2M* as a housekeeping gene has a critical impact on the interpretation of PCR data.^32^ In our assay, the dynamic quantification range (from 6 × 105 copies to 6 × 1010 copies) was satisfactory for clinical use. When this system was applied to clinical samples, we found that there was a statistically significant increase (p < 0.01) in the average *MRP1* expression level at the time of nonresponse or early relapse, compared with patients in remission. The *MRP1* expression level could be correlated with clinical response. This result is in agreement with observations by other authors who also found statistically significant increases in *MRP1* expression in relapsed leukemia patients.^5,16,22,33^ In addition, the mean expression level in relapsed samples is somewhat lower than in NR cases. This may be due to the nature of cancer cells and chemotherapy-induced events. Treatment induces rapid apoptosis of the sensitive cell fraction, leaving a small but substantial number of resistant cells.

As mentioned earlier, the relationship between *MRP1* expression and response to treatment in patients affected by leukemia is still controversial. We addressed the question of whether or not *MRP1* expression would be elevated in Relapsed and NR patients, thus offering a possible explanation for their relapse phenotype. Healthy individuals served as controls, such that their *MRP1* levels would be the base levels to which the *MRP1* levels of the patients would be compared. As we have reported, the *MRP1* levels in Relapsed and NR patients were significantly higher than those in the controls, thus suggesting that the lack of optimal response to drug treatment may be due to this elevated expression.

The patients in the CR group were also tested because the results from that group might have been revealing and interesting, if the *MRP1* levels in this group were also found to be high. This would have made the hypothesis that *MRP1* levels had a role in drug response at least less likely.

However, in all the AML patients in CR, the *MRP1* levels were similar to those in healthy controls. Therefore, it is possible that the observed CR phenotype is like this precisely because these patients’ *MRP1* levels never increased in response to drug treatment, and that this contributed towards the effectiveness of the treatment.

Although this correlation was obvious in AML patients, no meaningful correlation was observed between *MRP1* mRNA levels and response to chemotherapy in ALL samples. After we redefined the cutoff value according to the mRNA level in newly diagnosed patients, some of the samples that had previously been assumed to present overexpression rolled back to the normal group, but no change in the overall results was observed either in AML or in ALL patients. When we used the first cutoff value, *MRP1* overexpression was observed in the relapsed ALL patients but, according to the alternative cutoff, no cases of ALL in the relapsed or NR group showed overexpression. It seems that the basic amounts of *MRP1* mRNA in various types of leukemia are different and that attention must be paid to this phenomenon when defining the cutoff value. Hence, defining the cutoff according to the mRNA level in newly diagnosed cases in each disease could solve the paradox of meaningless overexpression of ABC transporter genes observed in cancer cells.^23^

According to our data, there was no clear relationship between *MRP1* expression and the patients’ gender, age, WBC count and CD34 expression. However, clinical parameters such as FAB subtype and cytogenetic risk group were found to be significantly related to the CR rate among AML patients. Genetic alterations could be detected in approximately 80% of *de novo* AML cases. These alterations are not only recognized as important initiating events in the process of leukemogenesis but also as indicators of clinical outcome. Deletion of the *MRP1* gene in AML patients with the 46, inv(16) karyotype was associated with a favorable effect on disease outcome.^33^ Similarly, in this study, we found four cases of AML with no *MRP1* expression, in which the cytogenetic group was favorable, including three cases with inv16 and one case with del 16. Our data suggest that changes at DNA level could contribute towards remission in AML and emphasize the role of *MRP1* in chemotherapy resistance. FAB subtype represents an additional relevant prognostic factor in relapsed AML cases. In our study, heterogeneity of *MRP1* expression among patients with different FAB subtypes was observed. Among the subtypes in AML cases, the mean *MRP1* expression in the M5 subtype (0.6415 ± 0.071) was significantly higher than in other subtypes (p-value = 0.001). The M5 subtype seemed to correlate negatively with Pgp function, without a better prognosis,^34^ so it is possible that *MRP1* overexpression is responsible for the MDR phenotype in the M5 subtype of AML patients.

## CONCLUSIONS

In summary, the mRNA level of *MRP1* was determined by quantitative real-time PCR in patients with acute leukemia. We showed that, in AML patients, the *MRP1* mRNA levels of the Relapsed and NR groups were significantly higher than those of the controls, thus suggesting that the lack of optimal response to drug treatment may be partly due to this elevated expression. We assessed the relationship between *MRP1* mRNA levels and other important clinical characteristics such as cytogenetic subgroups and FAB subtypes, and we showed that high expression of *MRP1* was correlated with FAB subtype and cytogenetic risk groups among AML patients.
